# Targeting p97 to Disrupt Protein Homeostasis in Cancer

**DOI:** 10.3389/fonc.2016.00181

**Published:** 2016-08-03

**Authors:** Pratikkumar Harsukhbhai Vekaria, Trisha Home, Scott Weir, Frank J. Schoenen, Rekha Rao

**Affiliations:** ^1^Division of Hematologic Malignancies and Cellular Therapeutics, Kansas University Medical Center, Kansas City, KS, USA; ^2^The University of Kansas Cancer Center, University of Kansas, Kansas City, KS, USA; ^3^Specialized Chemistry Center, University of Kansas, Lawrence, KS, USA

**Keywords:** ERAD, cancer, p97 inhibitors, proteotoxic stress, VCP, CDC48, ER stress

## Abstract

Cancer cells are addicted to numerous non-oncogenic traits that enable them to thrive. Proteotoxic stress is one such non-oncogenic trait that is experienced by all tumor cells owing to increased genomic abnormalities and the resulting synthesis and accumulation of non-stoichiometric amounts of cellular proteins. This imbalance in the amounts of proteins ultimately culminates in proteotoxic stress. p97, or valosin-containing protein (VCP), is an ATPase whose function is essential to restore protein homeostasis in the cells. Working in concert with the ubiquitin proteasome system, p97 promotes the retrotranslocation from cellular organelles and/or degradation of misfolded proteins. Consequently, p97 inhibition has emerged as a novel therapeutic target in cancer cells, especially those that have a highly secretory phenotype. This review summarizes our current understanding of the function of p97 in maintaining protein homeostasis and its inhibition with small molecule inhibitors as an emerging strategy to target cancer cells.

## Introduction

Cell division control protein 48 (CDC48), or p97, is a member of the type II AAA+ protein family of ATPases that participates in a diverse array of cellular activities (1–3). AAA+ proteins are evolutionary conserved class of proteins found in archaea, bacteria, viruses, and all eukaryotes. They typically assemble into hexameric complexes that utilize the energy of ATP-hydrolysis to facilitate macromolecular remodeling (4). Consequently, AAA+ proteins are involved in numerous processes including protein folding, DNA recombination, repair or replication, metal chelation, and proteasome-associated activities (2). p97 is an abundantly expressed cellular protein involved in the disassembly of protein complexes particularly, in proteasomal protein degradation, chromatin remodeling, autophagosome maturation, immune signaling, endoplasmic reticulum (ER) membrane fusion, and assembly of Golgi membranes (5). This review highlights the role of p97 in protein homeostasis and describes the consequences of its chemical or genetic inhibition in cancer cells.

**Figure 1:**
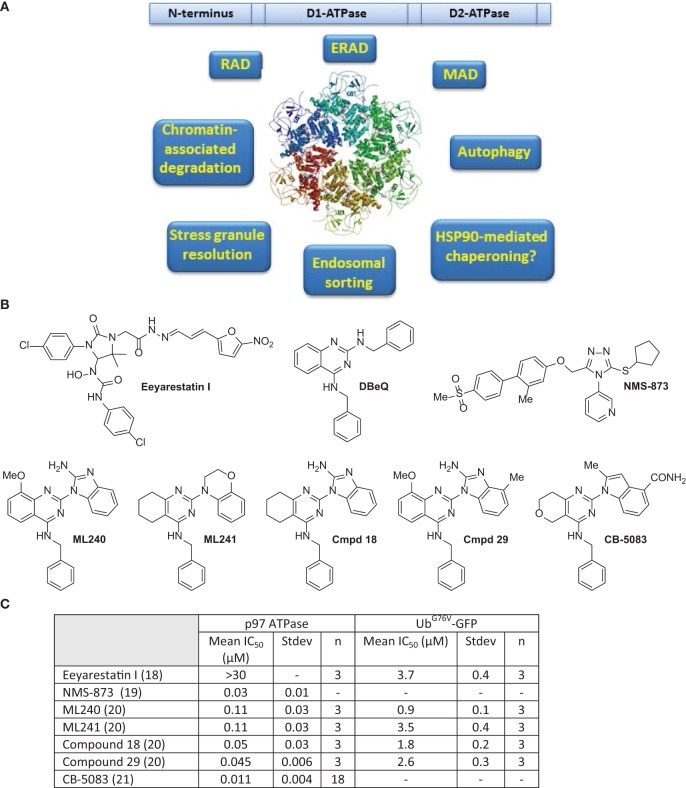
**(A)** Domain structure, ribbon diagram of p97 hexameric structure (11), and functions of p97 are summarized. **(B)** Select inhibitors of p97 ATPase. Chemical structures for Eeyarestatin I, DBeQ, NMS-873, ML240, ML241, compound 18, compound 29, and CB-5083. **(C)** Comparison of IC_50_ values for shown compounds for p97 in biochemical and cell-based assays as reported in Ref. (18–21).

p97 comprises two AAA domains called D1 and D2, as well as an N-terminal domain that is involved in substrate and/or adaptor molecule recognition (6–8). The C-terminal tail of p97 is fairly unstructured and usually terminates with a hydrophobic residue, a tyrosine, and variable amino acid residues. The C-terminus tail also binds numerous adaptors, thus increasing the repertoire of p97 interacting proteins (7). p97 assembles into a homohexameric, barrel-like structure, in which the D1 and D2 domains are stacked in a head-to-tail manner. The axial channel resulting from the hexameric organization of p97 is used to translocate proteins and/or nucleic acid substrates and aid in their turnover or remodeling (9–11). The cryo-EM structure of p97 reveals the upward displacement of the N-terminal domain upon ATPγS binding to D1 and D2 domains. These structural changes are essential for 97 activity and are blocked by the p97 inhibitor UPCDC3024 (Figure 1) (11). The D1 domain is important for mediating the assembly of p97 hexamers and has very low ATPase activity (4, 12). The D2 domain contributes to the major share of ATPase activity in p97 (13). However, a functional D1 domain and cooperativity between both D1 and D2 domains is necessary for optimal p97 function and cell growth (13–15). The linker region between the D1 and D2 domains has also been reported to be important for ATPase activity and asymmetric assembly of p97 (16). p97 interacts with its ubiquitinated substrates through a diverse array of ubiquitin adapters that contain the ubiquitin-binding domain (UBD), or p97-binding (UBX), or UBX-like (UBX-L) domain. It also employs numerous cofactors that aid in its cellular functions (17) (Table 1).

**Table 1:** **A partial list p97 interacting proteins and their associated functions**.

p97 interactors	Function
p47	Golgi and ER biogenesis (22)
	ERAD (17, 23)
gp78	ERAD (17)
Otu1	Substrate deubiquitination (17)
Ufd1p-Nlp4	ERAD (24), inhibition of golgi membrane fusion (25), and NF-κB2 activation (26)
UBXD1	Protein trafficking (27)
HDAC6	Clearance of polyubiquitinated protein aggregates (28, 29)
VIMP	ER shaping (30) and ERAD (31)
UBXN10	Ciliogenesis (32)
SVIP	Inhibition of ERAD (33) and autophagy (34)
HSP90	Regulation of HSP90 chaperone function (28)

## p97 and Its Role in Cellular Adaptations to Proteotoxic Stress

Tumors are “addicted” to oncogenic mutations such as gene amplification, loss of tumor suppressor function, or gain-of-function mutations. However, it has become increasingly clear that several non-oncogenic traits or “stress phenotypes” enable them to thrive in the hostile tumor microenvironment (35). Examples of the stress phenotype include replicative/DNA damage stress, proteotoxic stress, oxidative stress, and metabolic stress (36). Due to altered genomic copy numbers and the resultant synthesis of non-stoichiometric amounts of cellular proteins, tumors are experience heightened proteotoxic stress. The proteotoxic stress phenotype of tumors, in turn, makes them highly dependent on heat shock protein 90 (HSP90) chaperone function to promote protein folding and maturation (35, 37, 38). The following section highlights key aspects of p97-dependent cellular adaptions to proteotoxic stress. Other functions of p97, such as its chromatin-associated functions, etc., are beyond the scope of this review and are covered in many excellent publications (39, 40).

### HSP90-Dependent Chaperoning of Misfolded Proteins

p97 is a key player in the clearance of misfolded proteins in concert with the HSPs and the stress-inducible transcription factor, heat shock factor 1 (HSF1) (41). HSF1-induced HSPs (HSP90 and its co-chaperones (HSP70, HSP40, and HSP27) participate in the refolding and maturation of proteins. Misfolded proteins that cannot be refolded are polyubiquitinated and degraded by the ubiquitin proteasome system (41–43). p97 exists in a “repressive” complex with HSP90, histone deacetylase 6 (HDAC6), and HSF1. Accumulation of misfolded proteins in the cell and the ensuing proteotoxic stress disrupt the repressive complex. This, in turn, results in the activation of HSF1 by a process that involves its phosphorylation, trimerization, and nuclear translocation (28). Nuclear HSF1 activates the transcription of HSPs that promote protein refolding. The HDAC6 released from the complex binds to misfolded, polyubiquitinated proteins and facilitates their shuttling into peri-nuclear structures called aggresomes. p97 has been demonstrated to be involved in the HDAC6-dependent fusion of aggresomes with an autophagolysosomes – a vesicular body that promotes the clearance of ubiquitinated proteins and/or damaged organelles (2, 44, 45). By virtue of its segregase activity, p97 dissociates HDAC6-polyubiquitin complexes and determines the extent of HDAC6-dependent shuttling (and accumulation) or clearance of polyubiquitinated proteins by proteasomes or autophagosomes (29, 46). p97 is also required for the reformation of the HSP90-HDAC6-HSF1 complex, which occurs only when HDAC6 is not bound to polyubiquitinated proteins (47). The dissociation of HDAC6 from the repressive complex could potentially affect the acetylation status of HSP90, which negatively regulates HSP9 chaperone function (48, 49). The impact p97 inhibitors on HSP90 chaperone function has, however, not been studied.

### Unfolded Protein Response and ER-associated Degradation

Nearly thirty percent of mammalian proteins pass through the ER where they are folded, glycosylated, and assembled before they reach their final destination within or outside the cell (50). A series of glycosylation/deglucosylation steps as well as the unique oxidative environment in the ER promote the proper folding of membrane-resident and/or secretory proteins (51). Perturbations in ER function due to intrinsic and extrinsic signals, such as hypoxia, nutrient deprivation, deregulation of Ca homeostasis, enhanced rates of protein synthesis, etc., lead to the accumulation of misfolded proteins in the ER (52). These perturbations result in ER stress – a stress phenotype that is prevalent in both normal and malignant secretory cells (53, 54).

Unfolded protein response (UPR) is a cellular adaption that ensues following induction of ER stress. UPR activates three important signaling pathways: the inositol-requiring enzyme 1α (IRE-1α), PRKR-like ER kinase (PERK) kinases, and as the activating transcription factor 6 (ATF6) (reviewed in detail elsewhere) to restore proteostasis (55). Activation of UPR induces the phosphorylation of the transmembrane kinase IRE-1α, which catalyzes the splicing of X-box-binding protein (1) (XBP1) which, in turn, induces the transcription of HSP40 family members ERdj4 and the ER degradation enhancer, mannosidase alpha-like 1 (EDEM1), a protein required for ERAD (discussed below) (56). Activation of PERK phosphorylation induces the phosphorylation of eukaryotic transcription factor 2α (eIF2α), which halts overall protein translation but induces the translation of activating transcription 4 (ATF4) and other selected mRNAs. ATF4 also induces the pro-apoptotic transcription factor CCAAT/enhancer-binding protein (C/EBP) homologous protein (CHOP), which upregulates numerous apoptotic targets genes including BIM, death receptor 5 (DR5), and ER oxidase 1α (ERO1α) (57, 58). ATF6α is also a transmembrane ER protein, which is proteolytically processed in response to ER stress (59). ATF6α enters the nucleus to activate the transcription of chaperones glucose regulated proteins 78 and 94 (GRP78 and GRP94) as well as XBP1. Overwhelming amounts of misfolded proteins or prolonged ER stress activate the PERK pathway to upregulate CHOP as well as ATF4 transcription factors. These events promote ERO1α and protein disulfide isomerase-dependent production of reactive oxygen species (ROS) through protein disulfide oxidation, leading to oxidative stress and apoptosis (60).

Endoplasmic reticulum-associated degradation (ERAD) is a process by which misfolded proteins that cannot be refolded in the ER are retrotranslocated to the cytosol and degraded by the proteasomes (61–64). p97 uses its energy of ATP-hydrolysis to structurally remodel and extract ERAD substrates through the ER membrane into the cytosol. p97 works in association with a complex of ER membrane protein channels (also called dislocons) including Sec61 and Sel1L and adaptors, ubiquitin fusion degradation 1 (Ufd1)-nuclear pore localization 4 (Npl4) (24, 65, 66). Owing to the fact that misfolded proteins are “tagged” with mono or polyubiquitin before they can be degraded by the proteasome, another important component of the ERAD machinery is the ubiquitin ligase complex (65). Both luminal and membrane-associated misfolded ERAD substrates are ubiquitinated by E3 ubiquitin ligases (see below) (66, 67). The process of substrate dislocation and proteasomal degradation is tightly coupled in order to prevent the toxic aggregation of hydrophobic misfolded proteins in the cytosol. Non-glycosylated proteins that cannot be folded in the ER are also subjected to proteasomal degradation by ERAD through mechanisms that utilize GRP78 and SEL1L (68, 69). The last step in ERAD is the deubiquitination of ERAD substrates before they can begin their translocation into the proteasomes. p97 is, therefore, also found to be associated with several deubiquitinating enzymes (DUBs), including ataxin3, TOD1, Otu1, and VCIP135 (70).

### Ubiquitination and p97

p97 interacts either directly (e.g., Gp78 and HRD1) or through adaptors (e.g., UBXD7/Ubx5) to E3 ubiquitin ligases such as cullin RING ligases (CRL) to facilitate the ubiquitination of numerous ERAD substrates (67, 71, 72). CRLs comprise multi-protein complexes that include the cullin scaffold, a RING-domain protein, and a cullin-specific adaptor that recruits both ERAD and non-ERAD substrates for ubiquitination. CRLs make up more than 240 different ligases that enable p97 to ubiquitinate numerous substrates including hypoxia-inducible factor 1 (HIF1) α, RNA polymerase II large subunit (Rpb1), and Aurora B (72, 73). Additionally, p97 is involved in the polyubiquitin chain assembly of its substrates in concert with the E4 ubiquitin ligase, Ufd2 (74).

### Ribosome-Associated Degradation

mRNA with no stop codons also called non-stop mRNA or those with a polybasic tract, such as polylysine, which occurs during the translation of a poly(A) tail of a non-stop mRNA, are subjected to ribosome stalling and degradation of the nascent peptide (75). Accumulation of such aberrant peptides could potentially lead to reduced translation-competent ribosomes or produce aggregation-prone polypeptides. Two independent studies identified p97 as a component of the complex that is involved in the ubiquitination and proteasomal degradation of aberrant tRNA-linked nascent peptides from the ribosomes (76, 77). Additional components of this complex include the E3 ubiquitin ligase Listerin (Ltn1), translation-associated element (Tae2), and the ribosome quality control 1 (Rqc1) (76, 77).

### Mitochondria-Associated Degradation

The process of extracting misfolded peptides from mitochondrial outer membranes to facilitate their proteasomal disposal (MAD) also involves p97 (78). MAD employs the p97 cofactors, Ufd1-Npl4, for the translocation of damaged mitochondrial proteins. p97 also participates in the elimination damaged mitochondria by mitophagy, a process that is dependent on the E3 ubiquitin ligase Parkin and autophagolysosomal function (79). Recent studies in yeast have identified that Ufd3 or Doa1 and Npl4 both serve as substrate recruitment cofactors for p97. Doa1 is a member of the WD40 family of proteins that functions specifically in the degradation of mitochondrial substrates. Deletion of Doa1 does not affect the degradation of non-mitochondrial substrates nor does it sensitize cells to ER stress-inducing agents. The absence of Doa1, however, inhibits growth of cells in response to increased oxidative stress (80). An analogous role of the human homolog of Doa1, phospholipase A2 activating protein, in MAD or mitophagy has not been reported.

## p97 Inhibitors: From Hit to Lead

It is clear from the above description that p97 is involved in the clearance of misfolded proteins by affecting numerous protein homeostatic mechanisms. Other than the processes mentioned above, p97 is involved in resolution of stress granules (SGs), endosomal sorting, and chromatin remodeling, among other functions. SGs are cytoplasmic aggregates formed due to impaired translation initiation of mRNAs in complex with ribosomal subunits (77, 81, 82). Owing to its pivotal role in HSP90 chaperone function, ERAD, MAD, RAD, and autophagy, it is conceivable that p97 inhibition would induce the accumulation of misfolded proteins or toxic protein aggregates in the cell. Consequently, p97 inhibitors selectively target cancer cells because of their heightened sensitivity to agents that disturb protein homeostasis. Eeyrestatins (EerI and II) were the earliest identified p97 inhibitors that inhibited ERAD, albeit with low potency (83, 84). Several chemically distinct classes of p97 inhibitors have been identified since then (85–88). The compounds, described below, have led to the identification of a molecule (CB-5083) with drug-like properties, which is currently being tested in the clinic (21).

Given that the ATPase activity of p97 is essential for its segregase activity, high-throughput assays designed to measure the ATPase activity of recombinant p97 were utilized to screen compound libraries to identify p97 inhibitors (89). *N*^2^,*N*^4^-dibenzylquinazoline-2,4-diamine (DBeQ) was identified as a reversible, ATP-competitive p97 inhibitor that showed IC_50_ value of <10 μM in the ATPase activity assay (89). The inhibitory activity of DBeQ was further confirmed in cellular screens by monitoring the degradation of a p97-dependent substrate Ub^G67V^-GFP. DBeQ was also 50-fold less potent in inhibiting other unrelated ATPase activities, such as those of *N*-ethylmaleimide-sensitive factor (NSF) and the ATP-dependent chymotryptic activity of the 26S proteasome. Mechanistically, DBeQ upregulated CHOP and resulted in cell death suggesting that DBeQ-dependent inhibition of p97 resulted in a lethal ER stress response. DBeQ also induced autophagy, as evidenced by the enhanced lipidation of LC3 (microtubule associated protein light chain) or LC3-B (89, 90).

To develop more potent p97 inhibitors, two promising hits, DBeQ and *N*-benzyl-2-(2-fluorophenyl) quinazolin-4-amine, from the high-throughput screen were optimized through structure–activity relationship (SAR) studies (20). These studies resulted in the identification of two compounds ML240 and ML241 bearing different substitutions at the N2 position on distinct quinazoline core scaffolds (Figure 1) (20). Both ML240 and ML241 were ATP-competitive p97 inhibitors and possessed similar *in vitro* ATPase activity, with ML240 being modestly more potent than ML241 in the stabilizing Ub^G67V^-GFP. ML241 showed greater induction accumulation of polyubiquitinated proteins compared to ML240. However, ML240 induced more apoptosis and efficient poly(ADP-ribose) polymerase cleavage compared ML241. These findings suggest that there are mechanistic differences in the modes of action of ML240 and ML241. Among the two compounds, only ML240 induced the accumulation of LC3-B (a marker of early autophagosome formation or impaired autophagic maturation) and thus behaved similar to DBeQ (20, 90). ML240 also outperformed ML241 in its antiproliferative effects as assessed in the NCI-60 cancer cell line panel.

NMS-873, unlike the other p97 inhibitors described above, is an allosteric p97 inhibitor that induces the accumulation of polyubiquitinated proteins, leading to ER stress and inhibition of autophagosome maturation (19). NMS-873 is thought to disrupt the ATPase activity of p97 by binding to the linker domain between the D1 and D2 ATPase domains of p97, leading to the stabilization of D2-ADP bound p97. Consequently, NMS-873 showed reduced sensitivity to limited trypsin digestion, which is associated to its higher stability and potency (19). Both the ATP-competitive (DBeQ, ML240, and ML241) inhibitors as well as the allosteric inhibitor NMS-873 have differential effects on the D1 and D2 ATPase activity of p97. *In vitro* ATPase activity assays revealed that NMS-873 and DBeQ inhibit ATPase activity from both D1 and D2 domains. ML240 and ML241, however, seem to selectively inhibit the D2 domain of p97 (15). The inhibitory activity of ML240 and ML241 is also dramatically decreased by the presence of the p97 cofactor p47 (49- to 37-fold) compared to DBeQ and NMS-873 (2- to 6-fold) (15, 91). In contrast, cofactors p37 and Npl4-Ufd1 did not change the potency of ML240 and ML241 (92). These findings suggest that it is possible to develop p97 inhibitors that exhibit complex-specific inhibitory activities, which could be used to inhibit specific functions of p97.

While the compounds described, so far, in this review have excellent ATPase-inhibitory activities, they lack “drug-like” properties, thus making them unsuitable for *in vivo* studies. Utilizing DBeQ, ML240, and ML241 and cogeners such as Compound 18 and Compound 29 as the starting point, CB-5083 (1-[4-(benzylamino)-5H,7H,8H-pyrano[4,3-d]pyrimidin-2-yl]-2-methyl-1H-indole-4-carboxamide) was developed as a D2 domain selective, first-in-class p97 inhibitor with an IC_50_ of 11 nM (21). An in-depth analysis of the pathways affected by CB-5083 revealed that it affected the expression of mediators of the UPR (93). Significantly altered genes in response to CB-5083 treatment include *CHOP, DR5, HSPA5, HERPUD1, SEL1L, SYVN1, and EDEM1*. CB-5083 showed promising antitumor responses in colorectal, lung cancer, and plasmacytoma tumor xenografts as well as patient-derived xenograft models of colorectal cancer (93). The study also determined that resistance to CB-5083 was dictated by both mRNA and protein levels of p97 as well as possibly other cell-specific factors such as expression of EDEM1 and autocrine motility factor (AMFR). Additionally, activation of the mitogen-activated protein kinase (MAPK) pathway and phosphorylation of extracellular signal-regulated kinases 1 and 2 (ERK1/2) correlated with the activity of CB-5083. Owing to its promising pre-clinical activity, CB-5083 is being tested in the clinic against relapsed/refractory multiple myeloma and advanced solid tumors (NCT02243917 and NCT02223598).

## Potential Pitfalls and Advantages of p97 Inhibition

Activation of the IRE1α-XBP1 pathway in response to misfolded proteins in the ER induces ER chaperones that promote efficient protein refolding and promotes ERAD. However, XBP1 and PERK activation has also been reported to activate an epithelial–mesenchymal transition (EMT) phenotype in cancer cells (94–96). XBP1-induced EMT is associated with the upregulation of transcription factor Snail in breast cancer (96). Similar findings have also been reported in colorectal carcinoma (CRC) where the IRE1α-XBP1 pathway promotes proliferation and invasion of CRC cells (95). Consistent with these findings, siRNA-knockdown of p97 or treatment with Eeyarestatin I was reported to induce an EMT-like phenotype in lung adenocarcinoma cells (97). These studies indicate that p97 inhibition in cancer cells need to be considered with caution.

While studies have shown that the induction of ER stress in cancers promotes EMT and invasion, this can be considered as their vulnerability. This is because ER stress activation in cancers can potentially sensitize them to agents that accentuate ER stress (94). p97 inhibitors (EerI and DBeQ) have been reported to induce synergistic cell death in combination with the proteasome inhibitor bortezomib in mantle cell lymphoma and multiple myeloma (84, 98). Genetic knockdown of p97 inhibits cancer cell viability and synergizes with a wide variety of agents that induce DNA damage, growth inhibition, and cellular stress *in vitro* (19). Interestingly, no evidence of EMT-like phenotype has been reported for CB-5083 in *in vivo* models. Future studies to disrupt specific adaptor/cofactor-p97 associations will likely lead to the identification of novel p97 inhibitors with enhanced specificity and/or superior anticancer activity.

## Author Contributions

RR, SW, and FS conceptualized and prepared the mini review. PV and TH helped in manuscript preparation.

## Conflict of Interest Statement

The authors declare that the research was conducted in the absence of any commercial or financial relationships that could be construed as a potential conflict of interest.
